# Chronic Traumatic Encephalopathy as a Preventable Environmental Disease

**DOI:** 10.3389/fneur.2022.880905

**Published:** 2022-06-13

**Authors:** Michael E. Buckland, Andrew J. Affleck, Alan J. Pearce, Catherine M. Suter

**Affiliations:** ^1^Department of Neuropathology, Royal Prince Alfred Hospital, Camperdown, NSW, Australia; ^2^School of Medical Sciences, University of Sydney, Camperdown, NSW, Australia; ^3^College of Science, Health and Engineering, La Trobe University, Bundoora, VIC, Australia

**Keywords:** concussion, tau, punch-drunk, neuropathology, dementia, neurodegeneration

## Abstract

In this Perspective we explore the evolution of our understanding of chronic traumatic encephalopathy (CTE) and its relationship with repetitive head injury. As with many neurodegenerative conditions, there is an imperfect correspondence between neuropathology and clinical phenotype, but unlike other neurodegenerative diseases, CTE has a discrete and easily modifiable risk factor: exposure to repetitive head injury. Consequently, evaluation of the evidence regarding exposure to repetitive head injury and CTE risk should be undertaken using public or occupational health frameworks of medical knowledge. The current debate over the existence of CTE as a disease of concern is fuelled in part by immediate medico-legal considerations, and the involvement of high-profile athletes, with inevitable media interest. Moving beyond this debate has significant potential to address and reduce disease impact in the near future, and provide novel insights into mechanisms underlying abnormal protein accumulation in CTE and other neurodegenerative diseases.

## Introduction

Chronic traumatic encephalopathy (CTE) is a modern diagnosis with a long history. Reports on the long-term consequences of repetitive head injury (RHI) began appearing in the scientific literature around a century ago. At this time, fans of boxing had long noted that those boxers who sustained many blows to the head were prone to a deterioration informally known as “punch-drunk.” In the seminal medical description of punch-drunk in 1928, Martland described a spectrum of neurological and psychological decline (1). Serious consideration of the later-onset symptoms of RHI in boxers ensued in the mid-20^th^ century, leading to more informative descriptions of punch-drunk such as “dementia pugilistica,” “traumatic dementia,” and “traumatic encephalopathy.” The precise term “chronic traumatic encephalopathy” (CTE) was popularized in 1957 by Critchley as a “*suitable scientific alternative to punch-drunkenness*” (2). He coined the term to describe the associated clinical features, as well as the early pathological findings of “*gliosis, cortical atrophy, and internal hydrocephalus*” at autopsy (3). Since then, the term CTE has been common in medical parlance, used often as a clinical description, and used increasingly in place of previous analogous terms such as dementia pugilistica.

## Defining CTE

Despite its varied use as a clinical term in the historical literature, the term “chronic traumatic encephalopathy” means something quite different and definite in 2022: CTE is a post-mortem *neuropathological* diagnosis. It is defined by the accumulation of hyperphosphorylated tau (p-tau) in neuronal cell bodies and processes, located around small blood vessels, at the depths of cortical sulci (4). There are other pathological and immunohistochemical features that are often associated with CTE, but a definitive diagnosis absolutely requires the presence of perivascular neuronal p-tau (5).

In 2013, McKee and colleagues devised a consensus staging scheme for CTE, proposing four stages of increasing disease severity (I-IV) (6). These criteria have since been refined (by the same group) to include only two stages: “low CTE,” where neuronal p-tau, in the form of neurofibrillary tangles, is restricted to one or more neocortical areas, and “high CTE,” where similar p-tau pathology is more widely distributed throughout the neocortex as well as other brain regions such as CA1 and and CA4 of the hippocampus, amygdala, thalamus, and cerebellar dentate nucleus (4). This refinement in staging simplifies diagnosis, and improves consensus among neuropathologists.

Over the last 15 or so years there has been an exponential increase in scholarly reports of CTE, and a corresponding increase in media attention and public awareness. This began with the first autopsy reporting of CTE in an American football player who had suffered from cognitive impairment, mood disorder, and parkinsonian symptoms (7). This single neuropathology report in 2005 catapulted CTE into the public consciousness, and spurred hundreds of new studies into the association between repetitive head trauma [such as occurs in contact sports and the military (8, 9)] and later life neurological deficits and psychological symptoms.

## CTE is a Unique Neuropathological Diagnosis of Brain Disease

A host of neurodegenerative disorders are characterized by abnormal insoluble protein aggregates in specific cellular and subcellular localizations. The anatomical distribution of the pathology usually defines the clinical phenotype, as opposed to the specific protein involved. Aggregates of different proteins may thus present with a similar phenotype when they affect similar brain regions (10), and for this reason a definitive diagnosis, even for Alzheimer's disease (AD), requires examination of the brain after death. When autopsy is used to confirm the suspected cause of neurological decline, concomitant or surprising neuropathology is often identified as the cause of decline. For example, in a recent comparison of clinical and neuropathological diagnoses of 180 individuals, more than a third of cases had a clinical diagnosis that was different from the final (definitive) neuropathological diagnosis (11). Unfortunately, medical autopsy is not often pursued in modern times, so the true population incidence for any neurodegenerative disease is likely to be imprecise.

Tau aggregates are not exclusive to CTE, but rather are a common denominator in multiple neurodegenerative conditions, including AD, frontotemporal lobar degeneration diseases (FTLDs), and aging-related tau astrogliopathy (ARTAG) (12). ARTAG often co-occurs with CTE, particularly with increasing age. But the pathology of the two tauopathies is distinct: the pathognomonic lesion of CTE is p-tau aggregates in neurons and neurites ± astrocytes, while ARTAG is typified by astrocytic p-tau aggregates alone (12). Furthermore, the tau isoform in aggregates in ARTAG is 4R, but in CTE neuronal aggregates are composed of both 3R and 4R isoforms, like the p-tau aggregates in AD (13). CTE is thus a pathology that shares features with other neurodegenerative disorders, but the cellular distribution and chemical make up of p-tau in CTE is distinct.

Tau filaments can incorporate different isoforms of tau, and possess multiple ultrastructures, with different neurodegenerative tauopathies usually exhibiting different tau aggregate formations (14). Recent cryo-electron microscopy on p-tau aggregates from an individual with CTE uncovered a novel ultrastructure that is distinct from the conformation of p-tau in Pick's disease and AD, even though they share the same isoform stoichiometry (15). As well as a distinctive conformation, CTE p-tau aggregates also contain a yet unknown hydrophobic non-proteinaceous cofactor. Importantly, even though the p-tau in CTE is a distinct molecule from other tauopathies, it has been shown to have the same prion-like properties of infection and self-propagation as the p-tau in AD (16, 17).

## Traumatic Brain Injury Predisposes to Neurodegenerative Disease

Over the last few decades many studies have shown that a history of traumatic brain injury increases the risk for neurodegenerative disease [reviewed in Wilson et al. (18) and Bieniek et al. (19)], leading to a growing acceptance that repeated head trauma earlier in life provides a substrate for neurodegeneration later on (20). Such increased risk is seen across various neurodegenerative diseases including AD, where three meta-analyses have independently found that previous RHI confers an increased risk of AD (specifically in males), with relative-risk increases of two- to four-fold (21–23). Meta-analyses have also uncovered an association of previous RHI with increased risk of Parkinson's disease (24) and Amyotrophic Lateral Sclerosis (ALS) (25, 26), and two case-control studies have identified RHI as a risk factor for frontotemporal dementia (27, 28).

Contact sports such as boxing, rugby, and football are activities that carry a high risk of RHI. It is not surprising that the long-term effects of RHI have been best studied in American footballers. A large cohort study of 3,439 retired National Football League (NFL) professionals reported a significantly lower all-cause mortality in this group, which was principally attributable to their cardiovascular health (29). However, within this same cohort, mortality due to neurodegenerative disease was *three times higher* than that of the general population, and for AD, and ALS mortality was *four times higher* (30). The observed increases in neurodegenerative mortality with RHI were not only large but statistically robust. These findings were mirrored in a more recent retrospective study of 7,676 Scottish soccer players: compared to a matched non-playing control cohort of 23,000, mortality due to heart disease and other non-neurodegenerative disease was significantly lower in soccer players, but mortality due to neurodegenerative diseases was significantly higher (31). Furthermore, risk of neurodegenerative disease varies by position played, with goalkeepers having the lowest risk (32), presumably due to heading of the ball being lower among goalkeepers than players in other field positions.

It is clear that there is already a large (and still growing) body of evidence that strongly links prior RHI to increased risk of neurodegenerative diseases in later life. Moreover, in 2020, the Lancet Commission included traumatic brain injury as a definitive modifiable risk factor to target for dementia prevention (33). It should be noted however that autopsy confirmation of the clinical diagnosis was not pursued in most of the studies mentioned above. It is therefore possible that a proportion of the individuals clinically diagnosed with a neurodegenerative disease, for example AD, did not have AD, but rather CTE. Without proper neuropathological assessment we cannot know, but this possibility suggests that autopsy neuropathology should be included in cases of neurodegeneration where there is a known or suspected history of RHI.

## CTE Neuropathology is Closely Linked to RHI

Compelling evidence that links post-mortem CTE pathology with RHI comes from modern immunohistochemistry of the brains of ex-boxers originally studied by Corsellis et al. (34). In this revisitation of the neuropathology of “punch-drunk.” Goldfinger et al. (35) found that most (7/10) of those ex-boxers with a diagnosis of punch-drunk also had the typical pattern of abnormal p-tau accumulation characteristic of CTE. AD neuropathology was present in two of the 10 cases, one being concomitant with CTE pathology. Importantly, neither AD nor CTE pathology was found in those ex-boxers who had suffered from RHI but were not diagnosed as punch drunk. This indicates that CTE is not an inevitable consequence of RHI, and raises questions about modifying factors.

Very little is known about factors that might modify the risk of CTE apart from RHI. A single nucleotide polymorphism in the *TMEM106B* gene was identified as a potentially protective allele for CTE severity and neuroinflammation in an autopsy cohort of 86 CTE cases (36). This polymorphism was investigated as it is in high linkage disequilibrium with the strongest disease-modifying allele in TDP-43-associated frontotemporal dementia. The APOE ε4 allele appears to increase the chances of co-existent beta-amyloid deposition in CTE cases (37). Alcohol abuse may worsen symptomology in CTE, as it does for many other neurological conditions, however there is no evidence that alcohol itself affects tau deposition in the brain.

CTE pathology has now been found at autopsy in multiple cohorts of individuals with a history of RHI [reviewed in Wilson et al. (18)], and in most instances previous RHI is the only clear identifiable risk factor. An apparent exception, a recent study in which “mild” CTE was found in 6 of 8 men with no known history of RHI (38), appears to be misdiagnosis of ARTAG as CTE (39). Other available evidence indicates that CTE is very rare or absent in the general population outside of a history of head trauma. A recent post-mortem study of 310 individuals found that while ARTAG was frequently found in the general aging population (incidence of 38%), CTE was absent (40).

CTE severity correlates positively with both increasing age and increasing exposure to traumatic brain injuries over time. While we do not yet have the tools to monitor p-tau progression in people living with CTE, available evidence suggests that CTE is a progressive disorder. From a neuropathological standpoint this implies a sequential “spread” of tau pathology from a few to multiple neocortical foci, followed by medial temporal lobe regions, diencephalon, and brainstem. This p-tau patterning forms the basis of the consensus CTE staging scheme (4). CTE pathology tends to be more severe in older individuals, where it often is found along with other neurodegenerative pathology. In former NFL athletes, CTE stage is most closely correlated to the years of exposure to RHI (duration of football career), the number of years after retirement from football, and age at death (5). However, early stage CTE has been observed in older individuals (41), which suggests that CTE might remain indolent in some people.

## Symptomology of CTE

There is currently no clear set of criteria to diagnose CTE in life. It is however reasonable to ask whether there are specific functional deficits in a living individual later diagnosed with CTE post-mortem. Like many neurodegenerative conditions where disease confirmation occurs after death, efforts to ascribe a particular set of symptoms to CTE have been hampered by the necessarily retrospective design of research, and the confounding presence of co-morbid neurodegenerative pathology, which is particularly common in older age groups with later stage CTE (41). Nevertheless, there is compelling evidence linking CTE to neuropsychological symptoms based on studies of those individuals with “pure” CTE.

The ex-boxers described in the Corsellis series are again instructive and provide strong evidence for a link between CTE neuropathology and a spectrum of clinical symptoms. Those in the series later confirmed to have CTE exhibited an overlapping array of neuropsychological deficits encompassing behavioral, cognitive, and to a lesser extent motor problems (34, 35). No individual diagnosed with CTE was without symptoms and the majority (6/7) exhibited CTE as the *sole* neuropathology.

In a landmark report, Stern et al. collated clinical features from 36 individuals with sports-related RHI diagnosed with only CTE and no co-morbid neuropathology (42). Three of the 36 individuals were asymptomatic at the time of death, but the remainder of these individuals with “pure” CTE exhibited an array of neuropsychological symptoms including mood disturbances, loss of impulse control, impaired memory, headache, language deficits, visuospatial difficulties, executive dysfunction, and global cognitive decline. Stern et al. identified two clinical subgroups associated with CTE: one with younger age at symptom presentation with predominant behavioral symptoms, and another older age-at-onset more likely to present with cognitive impairment leading to dementia.

Subsequent clinicopathological studies of CTE [comprehensively reviewed in D'Ascanio et al. (43)], have supported this observation where there appears a continuum of symptoms associated with CTE stage in which changes in mood and behavior are usually the first indicators of disease. Most cases appear to exhibit progression of symptoms over time (44).

In 2014, Montenigro and colleagues proposed that features of CTE can be categorized as “traumatic encephalopathy syndrome” (TES), with mood, behavioral, cognitive, and movement subtypes (45). These TES criteria were not intended as diagnostic of CTE, but rather to facilitate CTE research. Later refinement of TES criteria has proven useful in clinical research (46), but appears more accurate in excluding a diagnosis of CTE, than positively identifying CTE (47).

## CTE Denialism

In their seminal description of punch-drunk neuropathology, Corsellis et al. conclude with the statement “*It is not suggested that this is a common sequence of events but that it has occurred occasionally can scarcely be denied*” (34). Unfortunately in 2022, despite the very large body of evidence linking RHI to later life decline, significant skepticism and confusion remains, particularly around CTE and its relationship to sports-related RHI (48). This confusion is exemplified by views expressed by those who seek to “first do no harm” with regards to reporting on CTE (49), and those who simply reject the premise of CTE as a “real” disease (50). These views do not seriously consider the possibility that RHI has the potential to result in serious damage, and that CTE may contribute significantly to this. The possibility that prominent, popular, and lucrative sports might pose a risk to health threatens personal and commercial interests.

The debate on CTE and RHI is reminiscent of controversies on tobacco use and lung cancer risk that were fostered by tobacco companies intending to protect their business, as recently discussed in an exchange of letters (51, 52). In current times, individuals who continue to play games with high rates of head trauma deserve to have the most complete available information regarding the long-term risks. Arbitrary dismissal of evidence for those risks will impede a fuller understanding that could lead to measures that mitigate or even eliminate the risk. There are acknowledged uncertainties around CTE, mainly in its clinical description, which is currently impeded by the inability to diagnose CTE in life. However, the strong association of CTE with RHI and a range of neuropsychological symptoms that appear progressive warrants widespread recognition, and intensive research investment.

## Moving Forward: CTE Recognition and Research

It could be argued that the burden of proof for CTE as a disease of concern has been placed far higher than for other neurodegenerative diseases. This is principally related to the proposed causal association with the environmental/occupational exposure to head injury, with obvious medico-legal and public health implications. Weighing of evidence in relation to CTE and RHI commonly occurs *via* the Oxford “Levels of Evidence” approach (53), where systematic reviews of randomized controlled trials provide the highest level of supportive evidence. But randomized controlled trials are simply infeasible with CTE, from both a practical and ethical perspective. The lack of such trials has led some to the conclusion that while there *is* an association with RHI, the evidence is “low-level” and hence may be in doubt (54). This sentiment is exemplified by the current international consensus statement on the management of concussion in sport (55), which states “*A cause-and-effect relationship has not yet been demonstrated between CTE and SRCs* (sports-related concussions) *or exposure to contact sports. As such, the notion that repeated concussion or subconcussive impacts cause CTE remains unknown*.” It is difficult to not interpret this statement as dismissive of CTE as a cause for real concern. Notably many of the authors of this consensus statement have professional involvement in lucrative sports, leading to strong (and potentially damaging) conflicts of interests (56).

In our opinion, weighing of evidence in relation to CTE and repetitive head injuries, like any environmental risk, needs to be performed in the framework of public and occupational health methodologies, independently of professional bodies that oversee risk management. As elegantly discussed by Brand and Finkel (57), even well-accepted causal associations in public health, such as smoking and lung cancer, still have unanswered scientific questions and encompass examples of disease incidence without exposure (lung cancer in non-smokers), as well as absence of disease despite exposure (inveterate smokers without lung cancer). None of these ambiguities negate the important causal association.

While no study has yet been performed that has precisely determined the prevalence of CTE in the general population, available evidence suggests that it is rare outside of the context of previous head trauma (40, 58). In professional American football, conservative modeling (adjusting for the most extreme selection bias), estimates the risk of CTE at ~10% (59). However, professional American footballers are not the only demographic at risk of CTE: any person who is exposed to repetitive brain injury, *via* any means, is at increased risk. With recent case studies reporting CTE pathology in sports outside of American football (60–63), and where community contact sports participation is high, there is a pressing need to quantify risk and then implement strategies that can best modify the risk. There is also a need to be alert to the potential for CTE in non-sports scenarios associated with RHI, particularly those that may not be so easily modifiable (such as domestic violence, combat military service, and medical conditions (such as epilepsy) associated with frequent head impacts. In our opinion, brain autopsy should be pursued when there is such a history, where possible, particularly if associated with neuropsychological symptoms.

Development of biomarkers and clinical criteria to aid CTE diagnosis in living individuals is also a pressing need (45). Advanced imaging techniques (64–66) and biomarker discovery studies (67, 68) are moving toward that goal. Large-scale prospective multimodality studies [such as DIAGNOSE-CTE (69)] are underway and hold great promise for detecting CTE during life. Together, these clinicopathological studies provide a platform for accelerating our understanding of CTE, and the mechanisms underlying CTE and its relationship to RHI.

## Conclusion

CTE is a neurodegenerative pathology closely associated with a history of repetitive traumatic brain injury. Currently CTE can only be diagnosed after death, but the living signs and symptoms of those harboring CTE are indicative of RHI-induced neuropsychological decline. In addition to the obvious public health implications of a disease linked to a modifiable exposure, understanding the pathophysiological mechanisms linking RHI and neurodegeneration has potential significance for our broader understanding of neurodegeneration in general. Likewise, our understanding of CTE will increase as we continue to incorporate principles established within research of other neurodegenerative diseases.

Dismissal or downplaying of the evidence for the long-term consequences of RHI in sport, or elsewhere, does nothing to advance our understanding of either CTE, or neurodegeneration more broadly. The costs incurred by ignoring, downplaying, or denying CTE are likely to be far greater than the costs of acknowledging, researching, and acting on this preventable environmental disease as a matter of urgency.

## Author Contributions

CS and MB wrote the manuscript with input from all authors. All authors agree to be accountable for the content of the review, contributed to the article, and approved the submitted version.

## Funding

This work was supported by NSW Health, Sydney Local Health District (CS, AA, MB).

## Conflict of Interest

AP currently receives partial research salary funding from Erasmus+ strategic partnerships program (2019-1-IE01-KA202-051555). AP has previously received partial research funding from the Sports Health Check Charity (Australia), Australian Football League, Impact Technologies Inc., and Samsung Corporation, and was remunerated for expert advice to medico-legal practices. The remaining authors declare that the research was conducted in the absence of any commercial or financial relationships that could be construed as a potential conflict of interest.

## Publisher's Note

All claims expressed in this article are solely those of the authors and do not necessarily represent those of their affiliated organizations, or those of the publisher, the editors and the reviewers. Any product that may be evaluated in this article, or claim that may be made by its manufacturer, is not guaranteed or endorsed by the publisher.
